# Dogs as a source of *Salmonella* spp. in apparently healthy dogs in the Valencia Region. Could it be related with intestinal lactic acid bacteria?

**DOI:** 10.1186/s12917-020-02492-3

**Published:** 2020-08-03

**Authors:** E. Bataller, E. García-Romero, L. Llobat, V. Lizana, E. Jiménez-Trigos

**Affiliations:** 1 Research Group Microbiological Agents Associated with Animal Reproduction (PROVAGINBIO), Department of Animal Production and Health, Veterinary Public Health and Food Science and Technology (PASAPTA), Facultad de Veterinaria, Universidad Cardenal Herrera-CEU, CEU Universities, Carrer Tirant lo Blanc 7, 46115 Alfara del Patriarca, València, Spain; 2 Grupo Sanidad de Rumiantes, Facultad de Veterinaria, Universidad de Murcia, C/ Campus Universitario 7, 30100 Murcia, Spain; 3 Servicio de Análisis, Investigación, Gestión de Animales Silvestres (SAIGAS), Facultad de Veterinaria, Universidad Cardenal Herrera-CEU, CEU Universities, C/Tirant lo Blanc 7, 46115 Alfara del Patriarca, Valencia Spain; 4 Wildlife Ecology & Health group (WE&H), Universitat Autònoma de Barcelona (UAB), Edifici V, Travessera del Turons, 08193 Bellaterra, Barcelona, Spain

**Keywords:** *Salmonella*, Dog, Prevalence, Zoonosis, Risk factors, Raw food

## Abstract

**Background:**

Although salmonellosis is considered one of the most important food-borne zoonotic diseases in Europe, close contact between dogs and their owners can also be a potential source of *Salmonella* spp. for humans. This study assessed the prevalence and antimicrobial resistance of *Salmonella* spp. in apparently healthy dogs in the Valencian Region, eastern Spain. Moreover, a macroscopic comparison of lactic acid bacteria in both *Salmonella*-positive and *Salmonella*-negative dogs was carried out.

**Results:**

Of a total of 325 dogs sampled, 6 (1.85%) were positive for *Salmonella* spp. with 3 different serotypes, Havana (3), Mikawasima (2) and monophasic Typhimurium (1). All isolates were susceptible to all antimicrobials tested except monophasic *S.* Typhimurium, which was resistant to ampicillin. Finally, macroscopic results revealed that lactic acid bacteria had higher heterogeneity in the *Salmonella*-negative dogs than in the *Salmonella*-positive dogs. Although the results in our study showed a low prevalence of *Salmonella* spp., raw food has been suggested as a risk factor for bacteria in dog faeces.

**Conclusions:**

Public awareness campaigns on good hygiene practices, especially after handling canine faeces or raw food, are necessary. Furthermore, to reduce the potential transmission of bacteria, dogs should be fed food that has been properly cooked, as raw or undercooked food can be a source of zoonotic pathogens. Moreover, further studies must be performed to determine the relationship between lactic acid bacteria and *Salmonella* spp. in dog faeces.

## Background

Salmonellosis is considered one of the most important foodborne zoonotic diseases in Europe [1]. In 2017, 91,662 confirmed human salmonellosis cases were reported by all state members in the EU, and eggs, egg products, meat and meat products contaminated with bacteria were the main sources of human infection [1]. However, companion animals can also be a source of *Salmonella* spp. for humans [2–6].

Dogs are one of the most important companion animals in the world [7]. It has been estimated that in Spain, there are more than 7 million pet dogs [8]. Close contact between dogs and humans has been recognized as a potential source of *Salmonella* spp. zoonotic infection [2, 4, 9–11]. In fact, the most frequent serotypes isolated from human gastroenteritis cases correspond with more prevalent serotypes in dogs [11, 12]. In addition, companion animals could be an important source of multidrug-resistant *Salmonella* spp. [13]. Therefore, pet dogs should be considered a public health risk [4]. Children, elderly and immunocompromised individuals have a higher risk of salmonellosis because they are more susceptible to infection [2, 11, 14, 15].

Since the 1970s, previous studies have reported the zoonotic transmission of *Salmonella* spp. from dogs [16]. Nevertheless, the prevalence of this bacterium in dogs varies considerably. First, dogs usually act as asymptomatic carriers, and they can shed one or more serotypes intermittently for more than 6 weeks [11, 17]. Moreover, dogs can harbour *Salmonella* spp. in the intestine and mesenteric lymph nodes without evidence of clinical signs [10, 11, 18]. Different studies have shown a wide prevalence of *Salmonella* spp. that oscillates between 0 and 79% [11, 12, 18–25]. This variability makes establishing the real prevalence among dog populations a challenge.

Other factors that have been reported to influence the prevalence of *Salmonella* spp. in dogs are the environment where animals live or contact with wild animals or other infected animals [11, 18, 26]. Additionally, animal feeding has been described as one of the main risk factors for the prevalence of *Salmonella* spp. in dogs [11]. Feeding dogs with raw food has also been related to the high prevalence of salmonellosis [12, 20, 21, 27, 28].

Another factor related to the increase in *Salmonella* spp. in animals could be associated with the alteration of normal microbiota, which protects the gastrointestinal tract from colonization by pathogens; alterations in the normal microbiota could provide a suitable environment for bacterial replication [29, 30].

As mentioned above, the prevalence of *Salmonella* serotypes in clinically healthy dogs varies notably and may even be different depending on the country [11]. As far as we know, no studies on the prevalence of *Salmonella* spp. in dogs have been performed in Spain. Therefore, the objective of this study was to determine the presence of *Salmonella* spp. in asymptomatic dogs housed in different environmental conditions in the Valencian Region (southern Spain). Moreover, the antimicrobial drug susceptibility of the isolates was determined, and macroscopic analysis of gastrointestinal lactic acid bacteria (LAB) in *Salmonella* spp.-positive and *Salmonella* spp.-negative animals was also evaluated.

## Results

### The prevalence of *Salmonella* spp. in apparently healthy dogs

The number of dogs sampled and the prevalence of *Salmonella* spp. is shown in Table 1. A total of 325 dogs housed in three different environments were examined. Rectal swabs were obtained from 85 dogs from households (26.2%), 84 dogs from animal shelters (25.8%), and 156 dogs from hunting kennels (48.0%). Of the 325 sampled dogs, 140 (43.1%) and 185 (56.9%) were males and females, and 49 (15.1%) and 276 (84.9%) were puppies and adult dogs, respectively.

**Table 1:** The prevalence of *Salmonella* spp. in apparently healthy dogs and variables included in the study

Variable Categories		No. of Dogs (%)	No. positive for *Salmonella* spp.	*Salmonella* Serotype (n)	X2 (p-value)
Dog population	Households	85 (26.2)	1	*S*. Mikawasima (1)	1.86 (0.39)
	Animal shelters	84 (25.8)	3	*S*. Havana (3)	
	Hunting kennels	156 (48)	2	*S*. Mikawasima (1) Monophasic *S.* Typhimurium (1)	
Gender	Males	140 (43.1)	3	*S*. Mikawasima (1) *S*. Havana (1) Monophasic *S.* Typhimurium (1)	0.12 (0.73)
	Females	185 (56.9)	3	*S*. Mikawasima (1) *S*. Havana (2)	
Age	Puppies	49 (15.1)	2	*S*. Havana (2)	1.59 (0.21)
	Adults	276 (84.9)	4	*S*. Mikawasima (2) *S*. Havana (1) Monophasic *S.* Typhimurium (1)	
Diet	Commercial	196 (60.3)	3	*S*. Havana (3)	0.27 (0.60)
	Home prepared	129 (39.7)	3	*S*. Mikawasima (2) Monophasic *S. Typhimurium* (1)	
Raw food consumption	Yes	36 (11.1)	4	*S*. Havana (3) Monophasic *S.* Typhimurium (1)	19.18 (< 0.01)*
	No	289 (88.9)	2	*S*. Mikawasima (2)	
Source of water	Bottled water	11 (3.4)	0	–	0.24 (0.89)
	Running water	269 (82.2)	5	*S*. Mikawasima (1) *S*. Havana (3) Monophasic *S.* Typhimurium (1)	
	Well water	45 (13.8)	1	*S*. Mikawasima (1)	
Contact with wild animals	Yes	178 (54.8)	2	*S*. Mikawasima (1) Monophasic *S.* Typhimurium (1)	1.13 (0.29)
	No	147 (45.2)	4	*S*. Mikawasima (1) *S*. Havana (3)	
Overall		325 (100)	6		

*p value ≤0.01 was considered significant

Regarding feeding, of the 325 dogs analysed, 196 ate commercial pet food (60.3%), and the remaining dogs were also fed home-prepared food (39.7%). Moreover, 36 dogs (11.1%) were fed raw food. The type of water source was also asked in the questionnaire; 11 dogs (3.4%) drank bottled water, while 269 (82.8%) drank running water and 45 (13.8%) drank from a water well. Finally, whether the dogs were in contact with other animal species was also asked in the questionnaire, and 178 dogs of 325 (54.8%) were in contact with wild animals. Independent of the environment where dogs lived, only 6 (1.85%) of the 325 rectal swabs analysed were positive for *Salmonella* spp. No significant differences were found between the percentage of *Salmonella* spp. and the age, sex, type of food, water source or contact with wild animals. However, the prevalence of *Salmonella* spp. was higher in animals that were fed raw food compared to those that did not eat raw food, and this difference was statistically significant (*p* value ≤0.01).

Serotypes isolated from dog rectal samples are also shown in Table 1. Serotyping revealed 3 different serovars: *S*. Mikawasima (*n* = 2); *S*. Havana (*n* = 3) and Monophasic *S.* Typhimurium (*n* = 1).

### Antimicrobial susceptibility of *Salmonella* isolates

The antimicrobial susceptibility of *Salmonella* serotypes is shown in Table 2. All serotypes were susceptible to all antimicrobials tested except monophasic *S.* Typhimurium, which was resistant to ampicillin.

**Table 2:** Antimicrobial resistance of *Salmonella* spp. isolated from dog rectal samples

*Salmonella* serotype (No. of isolates)	Antimicrobial susceptibility										
	AMP	CTX	CAZ	GM	ND	CIP	AZM	TGC	SXT	CT	C
S. Havana (3)	S	S	S	S	S	S	S	S	S	S	S
S. Mikawasima (2)	S	S	S	S	S	S	S	S	S	S	S
Monophasic S. Typhimurium (1)	R	S	S	S	S	S	S	S	S	S	S

*AMP* Ampicillin, *CTX* Cefotaxime, *CAZ* Ceftazidime, *GM* Gentamicin, *ND* Nalidixic acid, *CIP* Ciprofloxacin, *AZM* Azithromycin, *TGC* Tigecycline, *SXT* Trimethoprim-sulfamethoxazole, *CT* Colistin, *C* Chloramphenicol, *S* Susceptible, *R* Resistant

### Macroscopic LAB differences between *Salmonella-*positive and *Salmonella-*negative dogs

**Fig. 1:**
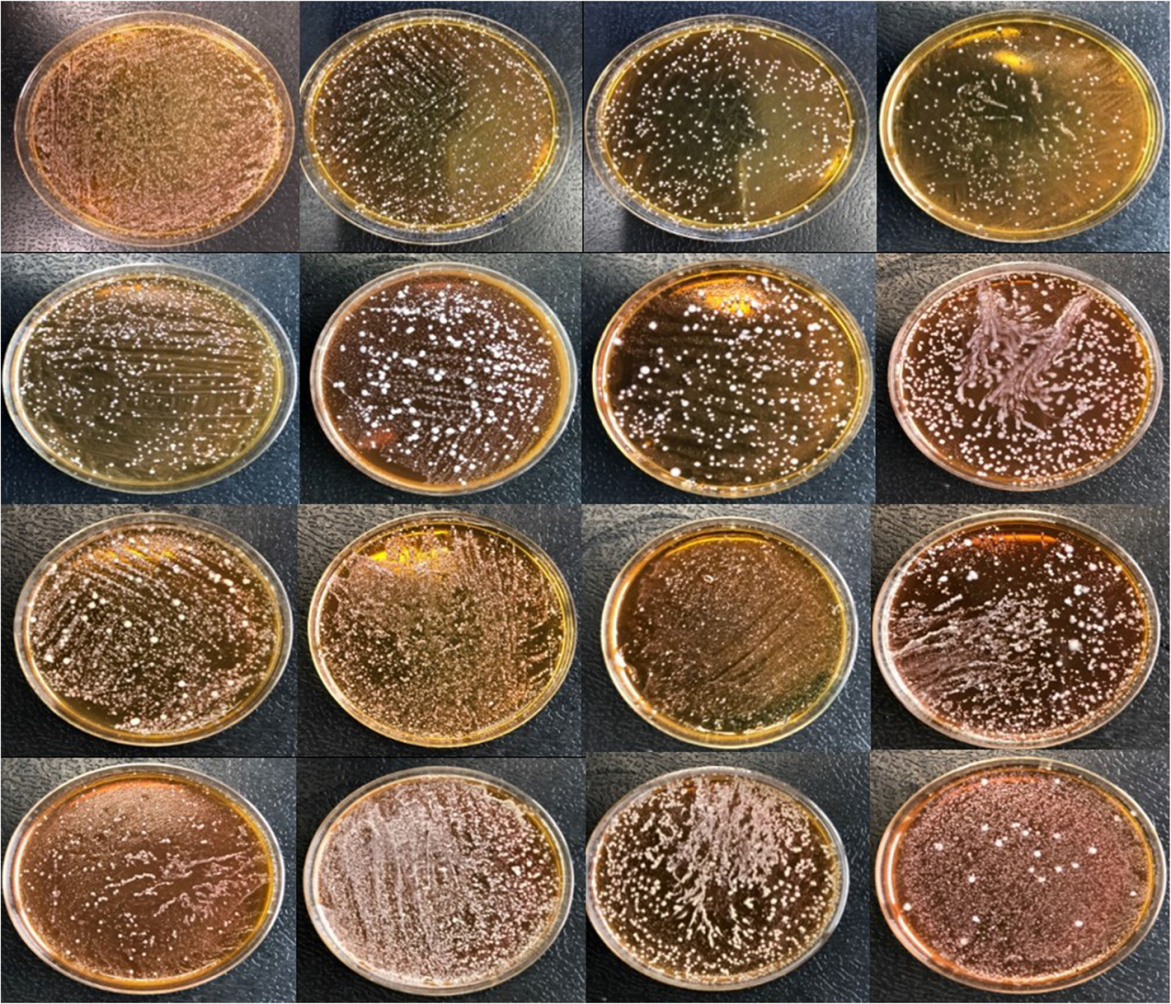
Macroscopic image of LAB from *Salmonella* spp.-negative dogs on MRS agar plates after 48 h of incubation at 37 °C under anaerobic conditions

**Fig. 2:**
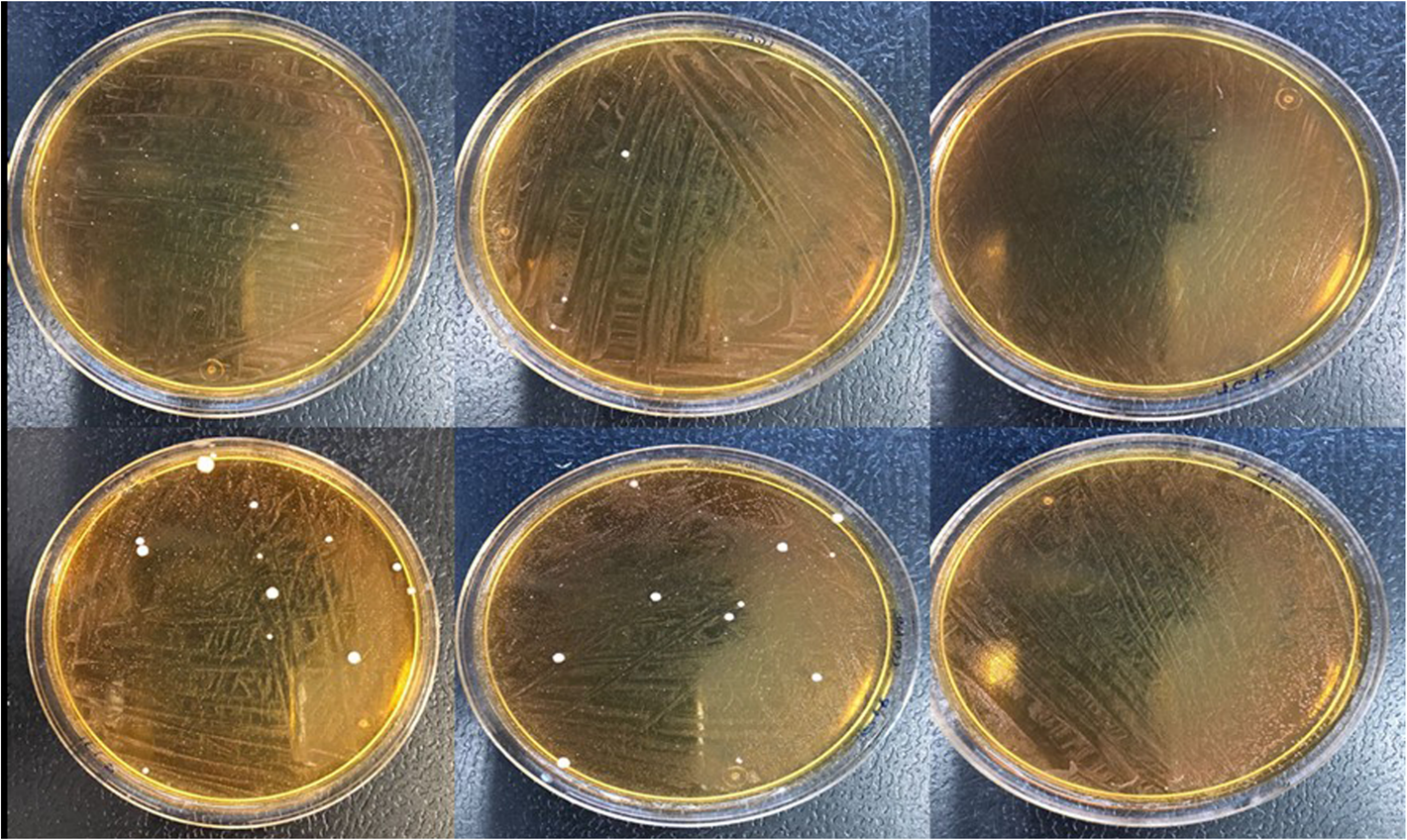
Macroscopic image of LAB from *Salmonella* spp.-positive dogs on MRS agar plates after 48 h of incubation at 37 °C under anaerobic conditions

LAB from *Salmonella*-negative dogs (*n* = 319) and *Salmonella*-positive dogs (*n* = 6) were grown on MRS agar. Macroscopic analysis was carried out, and subjective differences were observed. Figures 1 and 2 show the macroscopic profile of LAB from the *Salmonella*-negative and *Salmonella*-positive dogs, respectively. As an initial approximation, *Salmonella*-negative animals seemed to have more LAB than *Salmonella*-positive animals.

## Discussion

Although dogs may not play as large of a role in the transmission of salmonellosis to people as food, dogs have long been known to be asymptomatic carriers of *Salmonella* serotypes [31]. Their close relationship and frequent contact with humans, especially children, may make these animals a potential source of *Salmonella* spp. for humans and thereby represent an important public health issue [2, 10, 11, 18].

The isolation of *Salmonella* spp. from clinically healthy dogs has been reported to be between 0 and 79% [11, 21, 23, 24, 27]. The results obtained in the present study showed a prevalence of *Salmonella* spp. of 1.8% in apparently healthy dogs in Valencian Region (Southern Spain). Our results were in accordance with the literature. In most studies, the prevalence of *Salmonella*-positive dogs was below 3% [12]. Nevertheless, previous studies have shown that the prevalence of *Salmonella* in asymptomatic dogs can vary geographically [10, 11, 18, 19, 22, 23, 25]. Factors associated with these variations could be in part related to differences in the sample sizes, faecal sampling conditions and isolation and detection methods employed [11, 23]. In the present study, dogs were sampled only once. The low prevalence observed in our study could be related to the fact that only one faecal sample was collected, as intermittent shedding of *Salmonella* spp. has been well documented [11, 17]. However, rectal swab samples were employed in other studies to determine the presence of *Salmonella* in dog faeces; these studies observed similar results and a higher prevalence of *Salmonella* [32, 33].

The prevalence of *Salmonella* spp. in dogs has also been reported to depend on the immediate environment in which the animals live [11]. For example, the rates of isolating *Salmonella* from stray dogs have been reported to be significantly higher than those from household dogs [32]. In the current study, samples were collected from dogs housed in three different environments: households, animal shelters and hunting kennels. The results of our study are in accordance with Reimschuessel et al. (2017) [12]. They found that there was no significant statistical association with *Salmonella* status and living or exposure with other animals, age, exposure to water sources, hunting or performing sport activities. However, different studies have shown that the prevalence of *Salmonella* in dogs exposed to contaminated environments including hunting dogs or stray dogs is higher than that in household dogs [12, 23, 32, 33]; this result could be because dogs are more likely to roam free, scavenge, be in contact with carcasses or offals of wildlife and be fed raw or undercooked food [23, 33, 34].

As mentioned, differences in the prevalence of *Salmonella* can also vary depending on feeding practices [11]. Feeding pet raw meat-based diets (RMBDs) has received increasing attention in recent years [35]. Our results showed that 4 of 6 dogs positive for *Salmonella* were fed raw poultry carcasses. These results are in accordance with other studies that concluded that consuming raw diets increases the risk of carrying *Salmonella* spp. [12, 20, 21, 24, 27, 36].

Different studies have recorded that serovars found more frequently in dog faeces are similar to those found in humans [10, 12, 22, 32, 36, 37]. In the present study, three serotypes were isolated, monophasic *Salmonella* Typhimurium (16.7%), *S*. Havana (50%) and *S*. Mikawasima (33.3%), which have also been implicated in human salmonellosis [38–40]. All serotypes have been reported previously in dogs and humans [1, 16, 18, 39–45], and they have also been reported to be present in raw food [1, 39].

In our study, *S.* Havana were found in the faeces of *Salmonella*-positive dogs fed raw chicken carcasses. This serotype has been isolated previously from poultry products [46]. Pace et al. [38] determined a possible human infection caused by *S.* Havana from dog food contamination. *S.* Mikawasima has also been found in both wild boar from cattle-free and cattle-grazed areas in Spain [47], and in our study, *S.* Mikawasima was isolated from one hunting dog in contact with these species. Moreover, there has been an unusual increase in the number of *S.* Mikawasima infections in humans [39, 44]. Therefore, these results underline the potential public health risk of dog- and pet-contaminated foods [12, 22, 34].

In addition, a major problem in terms of both animal and human health is the emergence and increase in antibiotic resistance [34]. In fact, *Salmonella* has been listed by the World Health Organization (WHO) as one of the antibiotic-resistant priority pathogens and has become a worldwide health issue [48]. The results obtained in the present study showed that all serotypes isolated in our study were susceptible to all antibiotics tested. This is in accordance with other studies [11, 12, 49–51]. Only monophasic *S*. Typhimurium was resistant to ampicillin. Other authors have reported the isolation of resistant *Salmonella* strains from dogs [11, 12, 32, 52]. In addition, adding raw animal products to pet dog diets has been identified as a risk factor for the presence of antimicrobial-resistant *Salmonella* spp. [11, 53].

New strategies are urgently needed to manage antimicrobial resistant infections [34, 48, 53]. Among the various methods to reduce the use of antibiotics [54], probiotics are expected to be an alternative intervention measure to prevent bacterial infection in dogs [55]. To identify beneficial probiotic bacteria that inhibit pathogens, a better understanding of the dog microbiome is needed [56–58]. As a preliminary approach, we compared LAB in samples from both *Salmonella* spp.-positive and *Salmonella* spp.-negative animals, and it seems that macroscopic differences were observed. Commensal bacteria maintain the stability of the digestive tract and can help prevent intestinal infections by modulating the immune response and inhibiting certain pathogens such as *Salmonella* spp. that cause infections [58, 59]. The subjective differences observed at the macroscopic level in our study could suggest that some LAB may protect against the pathogen or, on the other hand, the presence of *Salmonella* spp. in dogs could inhibit beneficial LAB strains. Our preliminary results could be in accordance with other studies that observed that gut LAB in pigs inhibit the growth of *Salmonella* and prevent the adhesion of bacteria in the intestinal tract [60].

In that sense, further studies must be carried out to address the limitations of our study and analyse whether these differences could be related to *Salmonella* spp. infection. It would be interesting to identify whether some species of LAB in *Salmonella* spp.-negative dogs are able to inhibit *Salmonella* spp., which could explain the absence of infection. Host species specificity is considered a requirement for probiotics, especially as a therapy for GI disorders [58]. In that sense, the study of dog microbiota may lead to the discovery of new therapies that can act against pathogens such as *Salmonella* spp.

## Conclusion

In conclusion, the results obtained in this study indicate that apparently healthy dogs can act as subclinical carriers of *Salmonella* spp. in the Valencian region, eastern Spain. This could be significant to public health, as dogs are in close contact with humans. However, further studies must be performed because it is known that the shedding of *Salmonella* in dog faeces is influenced by several factors, such as diet, sampling procedures, and geographic area. Moreover, additional studies must be performed to determine the relationship between LAB and *Salmonella* spp. in dog faeces.

## Methods

### Sample collection and questionnaire

All animals were handled according to the principles of animal care published by Spanish Royal Decree 53/2013 [61]. Sample collection was carried out in the Valencian Region (eastern Spain) between October 2017 and June 2018. A total of 325 healthy dogs housed in three different environments were sampled (dogs were housed in households or animal shelters or were hunting dogs). Rectal specimens were collected using sterile cotton swabs (Cary Blair sterile transport swabs, Deltalab, Barcelona, Spain) by rotating the swab inside the rectum of the dog and then transported under refrigeration to the Laboratory of “Agentes microbiológicos asociados a la Reproducción Animal-ProVaginBIO”, UCH-CEU University, for *Salmonella* spp. isolation.

Data from each dog were also collected by a questionnaire developed for this study (Additional file 1) to determine the possible risk factors for *Salmonella* spp. infection, especially those related to the environment where animals were housed, the diet or type of food, contact with other animal species and the source of water. Other data were also included in this questionnaire, including gender and age. All questionnaires were completed and submitted together with the samples to the laboratory.

*Salmonella* spp. isolation, serotyping and determination of antibiotic susceptibility.

*Salmonella* isolation was performed according to ISO 6579: 2002 (Annex D) [62]. First, samples were preenriched for 18 ± 2 h at 37 °C ± 1 °C in 1:10 vol/vol buffered peptone water 2.5% (BPW; Scharlau, Barcelona, Spain). Then, 0.1 mL of the preenriched sample was transferred onto Semi-Solid Modification Rappaport Vassiliadis agar plates (MSRV; Difco, Valencia, Spain) and incubated at 41.5 °C ± 1 °C for 24–48 h. Suspicious growth on these plates was selected for inoculation onto Xylose–Lysine–Deoxycholate (XLD; Liofilchem, Valencia, Spain) and ASAP (ASAP, bioMerieux, Madrid, Spain) agar plates, which were incubated at 37 °C ± 1 °C for 24–48 h. After the incubation period, presumptive *Salmonella* colonies were selected, streaked onto nutrient agar plates (Scharlab, Barcelona, Spain) and incubated at 37 °C ± 1 °C for 24 ± 3 h. Then, a biochemical test using API (API-20, bioMerieux, Madrid, Spain) was performed to confirm the presence of *Salmonella* spp. Moreover, S*almonella* isolates were serotyped by the Ministry of Agriculture, Fisheries and Food Reference Laboratory (Algete, Madrid, Spain) according to the Kauffman-White-Le Minor scheme.

The antibiotics selected to test *Salmonella* spp. antimicrobial susceptibility were those set forth in Decision 2013/653 [63]: ampicillin (10 μg), cefotaxime (30 μg), ceftazidime (30 μg), gentamicin (10 μg), nalidixic acid (30 μg), ciprofloxacin (5 μg), azithromycin (15 μg), tigecycline (15 μg), trimethoprim-sulfamethoxazole (25 μg), colistin (10 μg) and chloramphenicol (5 μg). Antimicrobial susceptibility was tested according the European Committee on Antimicrobial Susceptibility Testing (EUCAST) guidelines [64]. The source for zone diameters used for interpretation of the test was http://www.eucast.org/clinical_breakpoints/. Zone diameters were interpreted and categorized as susceptible, intermediate or resistant according to the EUCAST clinical breakpoint tables.

### Lactic acid bacteria isolation

LAB isolation was carried out using the same rectal samples collected for *Salmonella* spp. isolation. Swabs were homogenized in BPW, and 100 μl was inoculated in the medium used for identification of LAB, especially *Lactobacillus* Man, Rogosa and Sharpe (MRS agar) (Scharlab, Barcelona, Spain) [65, 66], and incubated for 24–48 h under anaerobic conditions. After incubation, subjective macroscopic observation was carried out.

### Statistical analysis

Statistical analysis was performed with the statistical package R Commander and RcmdrPlugin. The associations between *Salmonella* occurrence; categorical factors were compared using Pearson’s χ2 test, and the confidence intervals for prevalence estimates were calculated using the Wilson score interval method. A *p*-value < 0.01 was reported as statistically significant.

## Supplementary information

**Additional file 1.** Questionnaire

## Data Availability

The datasets analysed during this study are available from the corresponding author on reasonable request.

## Abbreviations

ASAP: Chromogenic Culture Media for Rapid *Salmonella* Detection
BPW: Buffered Peptone Water
LAB: Lactic Acid Bacteria
MRS: Man Rogosa Sharpe
MSRV: Semisolid Modification Rappaport Vassiliadis
XLD: Xylose–Lysine–Deoxycholate
