# Supplementary material for: Dogs as a source of Salmonella spp. in apparently healthy dogs in the Valencia Region. Could it be related with intestinal lactic acid bacteria?
Source: BMC Vet Res. 2020 Aug 3;16:268. doi: 10.1186/s12917-020-02492-3 (PMC7398315; doi:10.1186/s12917-020-02492-3)
Supplement: Supplementary file 1 — Additional file 1. Questionnaire [file 12917_2020_2492_MOESM1_ESM.docx]

**QUESTIONNAIRE**

**PROJECT TITTLE:** *Salmonella* spp. prevalence in dogs from the Valencian Community, Spain.

| **Animal data** |
| --- |
| Name: |
| Breed: |
| Gender: |
| Age: |
| Group   - Housholds (Pets) - Animal Shelters - Hunting kennels |
| Diet or Type of food   - Dry food/commercial. - Home prepared - BARF DIET - Others:___________________________________________________________ |
| Source of water   - Bottled water - Running water - Well wáter |
| Contact with wild animals:   - Yes - No |
| Clincial history and treatments: |
| Vaccination and deworming program: |
